# Properties of Stereocomplex PLA for Melt Spinning

**DOI:** 10.3390/polym15234510

**Published:** 2023-11-23

**Authors:** Boris Marx, Lars Bostan, Axel S. Herrmann, Laura Boskamp, Katharina Koschek

**Affiliations:** 1 Faserinstitut Bremen, Am Biologischen Garten 2—Geb. IW3, D-28359 Bremen, Germany; bostan@faserinstitut.de (L.B.); herrmann@faserinstitut.de (A.S.H.); 2 Materials Engineering/Fibers and Fiber Composites Research Group, Faculty of Production Engineering, University of Bremen, Am Biologischen Garten 2—Geb. IW3, D-28359 Bremen, Germany; 3 Fraunhofer Institute for Manufacturing Technology and Advanced Materials IFAM, Wiener Straße 12, D-28359 Bremen, Germany; laura.boskamp@ifam.fraunhofer.de (L.B.); katharina.koschek@ifam.fraunhofer.de (K.K.); 4 MAPEX Center for Materials and Processes, University of Bremen, Bibliothekstraße 1, D-28359 Bremen, Germany

**Keywords:** PLA, stereocomplex, fibers, melt spinning, properties

## Abstract

Fibers made from biopolymers are one solution for conserving both resources and the environment. However, these fibers currently have limited strengths, which limit their use for textile applications. In this paper, a biopolymer stereocomplex poly(-lactide) (scPLA) formation on a technical scale of high-molecular-weight poly(D-lactide) (PDLA) and poly(L-lactide) (PLLA) is presented. This scPLA material is the basis for further research to develop scPLA yarns in melt spinning with technical strengths for technical application. scPLA is compared with standard and commercially available semi-crystalline PLA for the production of fibers in melt spinning (msPLA) with textile strengths. Differential scanning calorimetry (DSC) gives a degree of crystallization of 59.7% for scPLA and 47.0% for msPLA. X-ray diffraction (XRD) confirms the pure stereocomplex crystal structure for scPLA and semi-crystallinity for msPLA. scPLA and msPLA are also compared regarding their processing properties (rheology) in melt spinning. While complex viscosity of scPLA is much lower compared to msPLA, both materials show similar viscoelastic behavior. Thermal gravimetric analysis (TGA) shows the influence of the molecular weight on the thermal stability, whereas essentially the crystallinity influences the biodegradability of the PLA materials.

## 1. Introduction

Synthetic fibers produced by melt spinning are classified according to their strength [1]. Fibers with textile strengths of less than 50 cN/tex (450–680 MPa, depending on polymer density) are used in clothing, for example. Fibers with technical strengths of more than 50 cN/tex are used for more demanding applications (tire cord, ropes, composites, etc.). Standard thermoplastics, engineering thermoplastics or high-performance thermoplastics are used (e.g., polypropylene (PP), polyamide (PA) or polyether ether ketone (PEEK)) [2]. These conventional polymers have two major disadvantages [3]. They are synthesized from limited available petrochemical raw materials and they are not biodegradable. Thus, these polymers are challenging due to their huge impact on ecosystems. However, biopolymers such as poly(-lactide) (PLA) are made from renewable raw materials and they are biodegradable [4]. The global production volume of PLA grows exponentially and was around 190,000 tons in 2019 [5]. PLA is also the subject of intensive research regarding its properties, usability, productivity and substitute [6].

PLA exists in the variations poly(D-lactide) (PDLA), poly(L-lactide) (PLLA) and poly(DL-lactide) (PDLLA) [7,8]. The two enantiomers PLLA and PDLA are semi-crystalline polymers with melting temperatures (*T_M_*) of about 180 °C. By forming PDLLA copolymers of PDLA and PLLA, *T_M_* decreases and even an amorphous PLA can be produced. Thus, *T_M_* of PLA varies between approx. 130 and 180 °C. PLA for fiber production can be purchased from e.g., NatureWorks or TotalEnergies Corbion. The maximal strength of PLA yarns made from these granules is about 40 cN/tex (500 MPa) [9,10,11,12,13]. Due to their textile strength, PLA yarns can be used only in textile applications (wipes, sports fashion items, blankets, etc.) [14] and PLA is classified as a standard thermoplastic [2]. PLA for the production of fibers with technical strengths and thus for technical applications is currently not available on the market.

One promising method for the formation of PLA fibers with technical strengths is stereocomplex PLA (scPLA), first reported by Ikada et al. [15]: Pure stereocomplex (SC) was formed in solution by blending PDLA and PLLA at a racemic mixture. With the formed SC crystal structure, *T_M_* of PLA rises to around 230 °C through van der Waals forces between the two different helical chains. They also proved that SC can be formed from the melt [16]. Although amorphous, semi-crystalline and stereocomplex PLA material structures differ in degradation [17], the results of different research studies are contradictory [18]. However, scPLA has better thermal, mechanical and thermo-mechanical properties than semi-crystalline PLA [19,20,21]. SC therefore has the potential to transform PLA into an engineering thermoplastic. Despite different processing methods (melt processing, additive processing and solution casting) for the formation of scPLA [22] and despite extensive scientific investigations [23], no scPLA material for fibers with technical strengths is available on the market today.

In this work, we present the properties of an scPLA material, formed on a technical scale. This allows the development of PLA fibers with technical strengths in melt spinning. The process using a twin-screw extruder is explained and the particle size of the precipitated scPLA is analyzed. scPLA is compared to a common semi-crystalline PLA for fiber production with different methods: Thermal properties are determined using differential scanning calorimetry (DSC) and the crystal structure is analyzed using X-ray diffraction (XRD). Viscosity behavior is analyzed with rheology measurements. The thermal stability of the materials is examined by thermal gravimetric analysis (TGA). The biodegradability is evaluated by investigating the degree of decomposition under simulated compost conditions.

## 2. Experimental

### 2.1. Materials

PLA materials in granular form are purchased from NatureWorks “Ingeo” (Minneapolis, MN, USA) and TotalEnergies Corbion “Luminy” (Gorinchem, The Netherlands), respectively. Information about the lactide content, molecular weight and polydispersity index are given in Table 1. The high molecular weights of these PLA materials [24] are essential for more demanding applications. All three materials have a density of 1.24 g/cm³ and a glass transition temperature (*T_G_*) of 55 °C to 60 °C. PDLA and PLLA are the starting materials for scPLA formation. Before blending, PDLA and PLLA are dried for 24 h under a vacuum at 60 °C. The comparative material msPLA is a semi-crystalline PLA processed by melt spinning. Fibers made of this msPLA possess a maximum strength of 53 cN/tex (660 MPa) for a single filament according to the data sheet. The strength of the associated fiber yarn is lower due to inhomogeneities [25] and is about 40 cN/ tex (500 MPa) [9,10,11,12] as mentioned above. Potential applications for these msPLA fibers are staple fibers, nonwovens, agricultural wovens, articles for household disposal, textiles, continuous filaments, or spunbond fabrics according to the supplier.

### 2.2. scPLA Formation

During scPLA formation, PDLA and PLLA are fed in a nitrogen atmosphere to a twin-screw extruder (Leistritz) by two granule feeders (Coperion). The mass throughput (ṁ) for each material PDLA and PLLA is 1 kg/h. Hence, the twin screw extruder with twelve heating zones mixes the two materials continuously at a total ṁ of 2 kg/h and thus on a technical scale. The screw diameter (*d_S_*) is 18 mm and the cylinder length (*l_C_*) 48·d_S_. The screw rotates with a speed (*n_S_*) of 200 1/min. Since the scPLA formation presented in this study is a low-temperature blending [28,29], the extruder temperature (*T_E_*) is above *T_M_* of PLLA and PDLA and below *T_M_* of scPLA. Due to this temperature range, the scPLA precipitates as a powder.

### 2.3. Measurements

The precipitated scPLA powder was analyzed by image analysis (FibreShape automatic, V6.1.2f IST AG, Vilters, CH) [30]. A sample of more than 3000 particles was dispersed on the transparent sampling foil. The foil was transported through an Epson V700 Photo flat-bed scanner with a resolution of 1200 dpi. For evaluation, a powder shape algorithm set was used.

The thermal behavior of the PLA materials was analyzed using DSC (TA Instruments Q2000, New Castle, DE, USA) according to DIN EN ISO 11357-1 [31]. Weighed DSC samples were heated and cooled in a nitrogen environment at constant rates of 10 K/min. The degree of crystallization (*X_C_*) was calculated from the ratio of the melting enthalpy (*ΔH_M_*) and the enthalpy of fusion of a 100 % crystalline structure (*ΔH_M,100%_*), see Equation (1). *ΔH_M,100%_* = 93.1 J/g was used for msPLA [32] and *ΔH_M,100%_* = 146 J/g for scPLA [17].

(1) $$X_{C}=\frac{{∆H}_{M}}{{∆H}_{M,100\%}}\cdot 100\%$$

XRD experiments were performed on a D8 Discover diffractometer (Bruker, Billerica, MA, USA) using Bragg–Brentano geometry equipped with a Cu tube (kα 1.541 Å, 40 kV, 40 mA). The fixed divergence slit used is 0.12°. Continuous scans from *2θ* = 5° to 30° were performed at a scanning rate of 0.015°/s. The samples were pressed into thin pallets using hydraulic facilities.

The rheological cooling and heating behavior of the polymers was determined using a plate–plate rheometer (TA Instruments AR 2000ex, New Castle, USA) according to DIN EN ISO 6721-1 [33] at constant heating and cooling rates of 5 K/min. The elongation was 1%, the gap was 1 mm, the frequency was 5 Hz and the plate diameter was 25 mm.

TGA (TA Instruments Q5000 IR, New Castle, USA) was performed according to DIN EN ISO 11358 [34]. Weighed TGA samples were heated to 500 °C at 10 K/min in a nitrogen environment. At the beginning of each measurement, the temperature was kept constant at 60 °C for 60 min to dry the sample.

To investigate biodegradability according to DIN EN ISO 20200 [35], all raw materials were first dried for 24 h at 60 °C under a vacuum and then molded into forms (25 mm × 25 mm × 5 mm). The forms were stored in a bioreactor (3L) with the compost consisting of 40% sawdust, 30% rabbit feed, 10% ripe compost, 10% corn starch, 5% sugar, 4% corn oil and 1% urea. Distilled water was added in a 45:55 ratio for 94 days at a temperature of 58 °C in a climate chamber. After specific times, water was added to maintain the relative humidity in the compost and the samples were taken out for analyzing. The biodegradation test was stopped after 94 days, the cap of the bioreactors was removed and all samples were dried together with the compost for an additional 6 days at 58 °C.

## 3. Results and Discussion

The particle size, DSC, XRD and rheology of scPLA has already been compared with the starting materials PLLA and PDLA [36]. Therefore, msPLA and scPLA are considered for this analysis. TGA and degradation tests are presented for all four materials: PLLA, PDLA, msPLA and scPLA.

### 3.1. Particle Size Analysis

**Figure 1:**
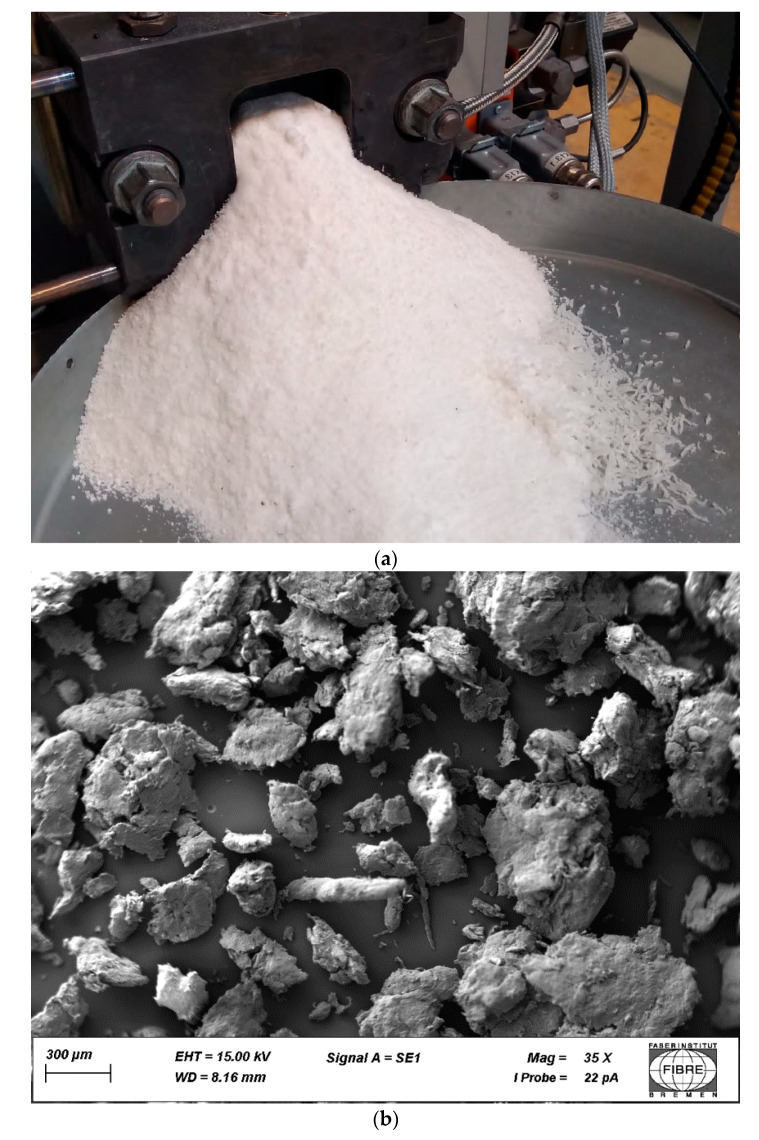
scPLA precipitation (**a**) and SEM image of scPLA powder (**b**).

Figure 1a shows the precipitation of scPLA during its formation. It is clear that powder precipitation is dependent on the ṁ, n_S_ and T_E_. At a higher T_E_, instead of the powder a melt is extruded and further processed in the classic way of blending [37]. The particle size as shown in Figure 1b can be influenced mainly by n_S_ and thus by the retention time of PDLA and PLLA within the extruder. The results of the particle size analysis of 3143 formed scPLA particles can be found in Table 2. The mean particle size is 493 µm. In total, 50% of the particles have a size of 423 μm, whilst 10% of the particles are smaller than 222 μm and 10% of the particles are bigger than 774 μm. Regarding the further processing of scPLA in melt spinning, the particle size distribution is important to know for the feeding of the extruder [38].

### 3.2. Thermal Properties

**Figure 2:**
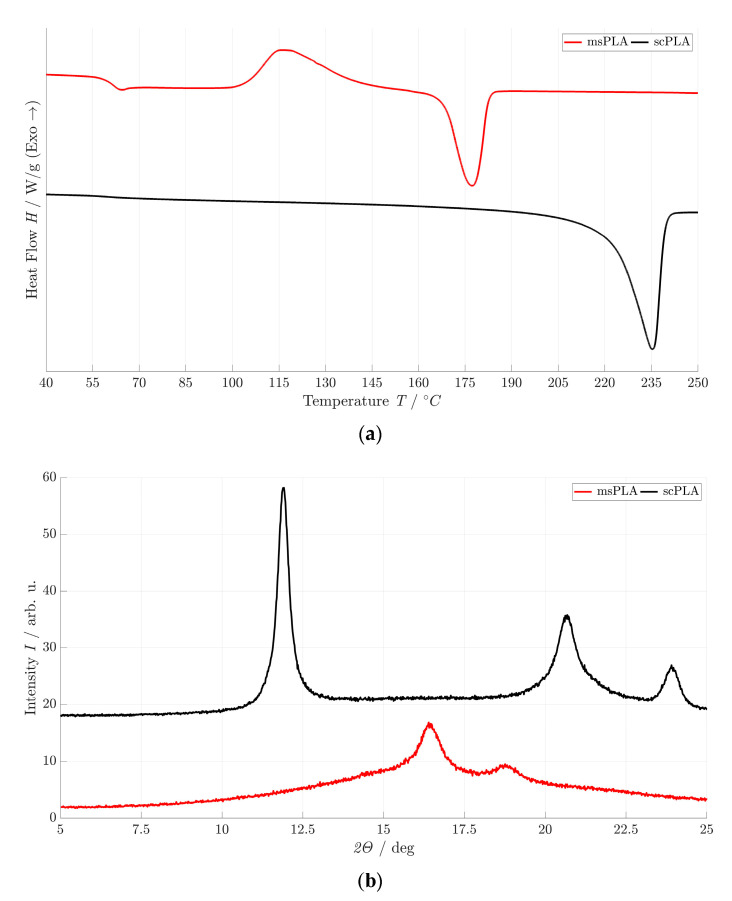
DSC thermograms (**a**) and XRD patterns (**b**) of msPLA and scPLA.

Figure 2a depicts the DSC curves of msPLA and scPLA. As with polyester (PET) [39], post-crystallization can also be seen with msPLA. The homocrystallites (HM) of msPLA post-crystallize at 116.1 °C. The corresponding crystallization enthalpies are shown in Table 2.

It can be seen that the post-crystallization of the semi-crystalline msPLA polymer passes directly into endothermic peaks at 178.0 °C as already reported by Ikada et al. [15]. The DSC thermogram of the scPLA curve shows only a single endothermic peak at 235 °C without any exothermic peak (post-crystallization). This increase in *T_M_* by 55 °C is caused by precipitation at a low temperature of PDLA and PLLA. The melting of scPLA can be clearly attributed to the stereocomplex (SC) crystallite [17]. The degree of crystallization (*X_C_*) of scPLA is 59.7% and significantly higher than that of msPLA (50.5%); see Table 1. *T_M_* and *X_C_* of scPLA presented in this paper are also higher than the results previously obtained by low-temperature approaches [28,29]. We assume that the use of the twin screw extruder favors the formation of the SC crystals due to more friction: kinetic energy in the form of shear enables the improvement of crystallization kinetics [40]. The higher the shear rate, the higher the crystallization rate and the content of SC crystals. Similarly, a reduction in shear temperature due to suppressed chain relaxation during shearing of the mixed melt results in significantly more SC crystals [41]. This is equally true for high molecular weight PLLA/PDLA mixtures [42].

### 3.3. XRD Patterns

scPLA and msPLA show different crystal structures, which can be clearly identified in the XRD patterns shown in Figure 2b. HM crystals (msPLA) and SC crystals (scPLA) can be clearly distinguished. The XRD patterns of msPLA exhibit two main diffraction peaks at *2θ* values of 16.5 and 18.5 assigned to the (200)/(110) reflection and the (006) reflection [43]. For scPLA, the observed maxima are at *2θ* values of 12°, 20.5° and 24° with the reflections (110), (300)/(211) and (220), respectively. This corresponds to the pure SC crystal structure [16,44]. Table 2 shows the *2θ* values of msPLA and scPLA.

### 3.4. Rheological Properties

**Figure 3:**
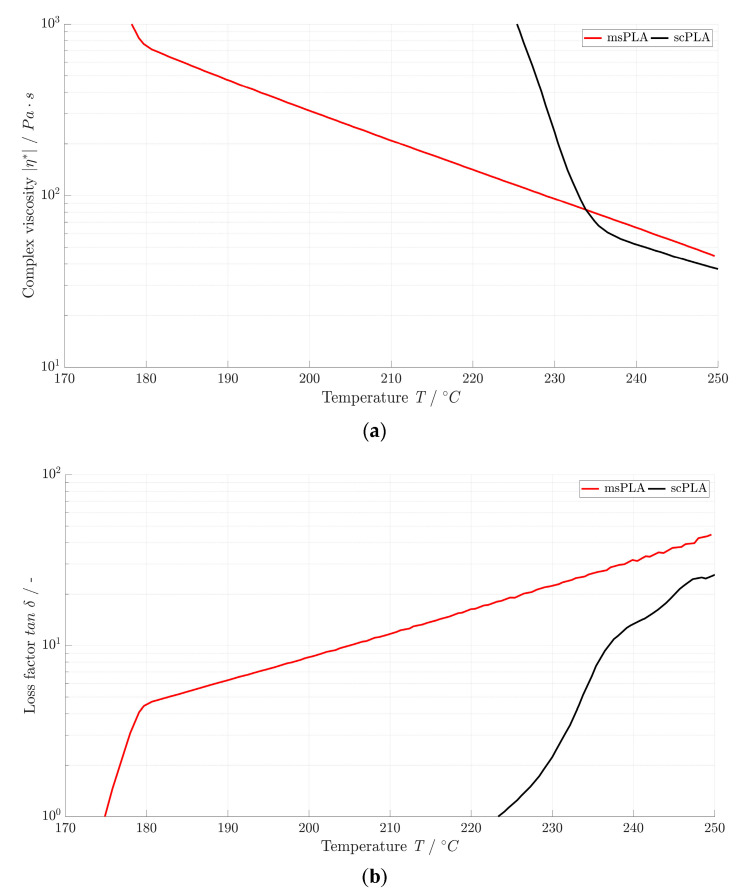
Complex viscosity (**a**) and loss factor (**b**) of msPLA and scPLA.

Figure 3a shows the complex viscosity |η*| of msPLA and scPLA depending on temperature. The recommended maximum processing temperature is 250 °C due to the thermal degradation of PLA [17]. Typical characteristics of two crystalline polymers (HM and SC) can be seen, since they melt as soon as the crystallite melting temperature is exceeded [45]. Between 180 °C and 250 °C, |η*| of msPLA lie in the range from 750 Pa·s to 45 Pa·s. This is in the range of values as for the melt spinning of PET (|η*| = 1000 to 250 Pa·s) [1]. It can be seen that scPLA is completely melted at 235 °C (|η*| = 60 Pa·s) and that |η*| decreases to 40 Pa·s at 250 °C. So, |η*| of scPLA is much lower at favored spinning temperatures than that of msPLA and similar to the viscosities of spinning solutions [1]. This means there are challenges in the extrusion of the material with regard to pressure control. Likewise, the ratio of the length and diameter of the nozzle holes in the spinneret must be redesigned. Figure 3b depicted a loss factor *tan δ* of msPLA and scPLA. With increasing temperature, the *tan δ* of both materials becomes larger: The elastic component decreases and the viscous component increases [45]. Within the molten state, the extreme values of *tan δ* for msPLA rises from 4.5 at 180 °C to 45 at 250 °C. These values for *tan δ* for msPLA in this temperature window correspond to the spinnability of PET, which is given for *tan δ* > 5 [46]. The *tan δ* of scPLA ranges between 7 at 235 °C and 26 at 250 °C. Thus, spinnability is also given.

### 3.5. TGA Results

**Figure 4:**
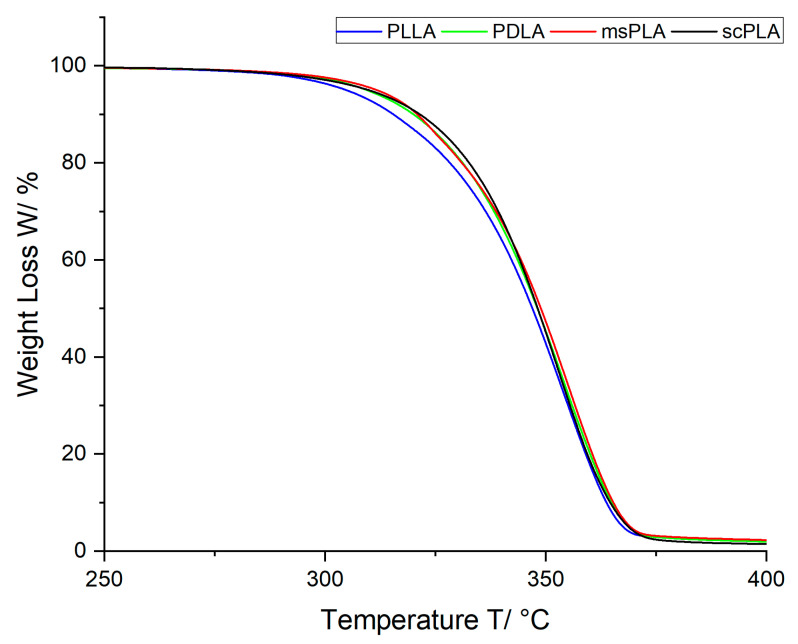
TGA results of PLLA, PDLA, msPLA and scPLA in granular/powder form.

The TGA plots of PLLA, PDLA, msPLA and scPLA in granular/powder form can be seen in Figure 4a. The temperatures at different weight losses are given in Table 3. The four curves are almost identical and no significant difference can be observed. These findings are consistent with the literature [25,40,41]: It is assumed that thermal degradation is essentially determined by the molecular weight and less by the crystal structure. As Table 1 shows, the molecular weights of the materials used are in the same range. Thus, as is the thermal degradation. The weight loss of PLLA, which starts at lower temperatures, can be explained by the significant lower degree of crystallization of 21.6% [36] compared to the other materials PDLA, msPLA and scPLA. These materials have a degree of crystallization of more than 40% (see [36] and Table 2).

### 3.6. Degradation under Compost Conditions

The effect of stereocomplex formation and crystallinity on the overall degradation rate under controlled aerobic compost conditions of PLLA, PDLA, msPLA and scPLA was conducted according to DIN EN ISO 20200. The process requires the use of a standardized and homogeneous artificial solid waste. The artificial waste is of constant composition and free of any unwanted plastics that could be misidentified as test material at the end of the experiment and thus change the final assessment. The bioreactors are small (about 3 L) in analogy to the amount of artificial waste to be composted.

Three specimens of each sample were placed in the reactor and recovered at different times to analyze the visual effects, gravimetric weight loss as well as TGA; see above. PLA usually undergoes a two-step degradation process. In the first step, water migrates into the sample causing hydrolysis of PLA by the chain scissions of ester groups. As soon as the molecular weight is reduced, the second process starts. The microorganisms start to assimilate the formed oligomers and lactic acid. This is also the reason why, among environmental conditions, polymer characteristics such as composition, crystallinity, molecular weight and additives also strongly effect the degradation rate [18,47]

**Figure 5:**
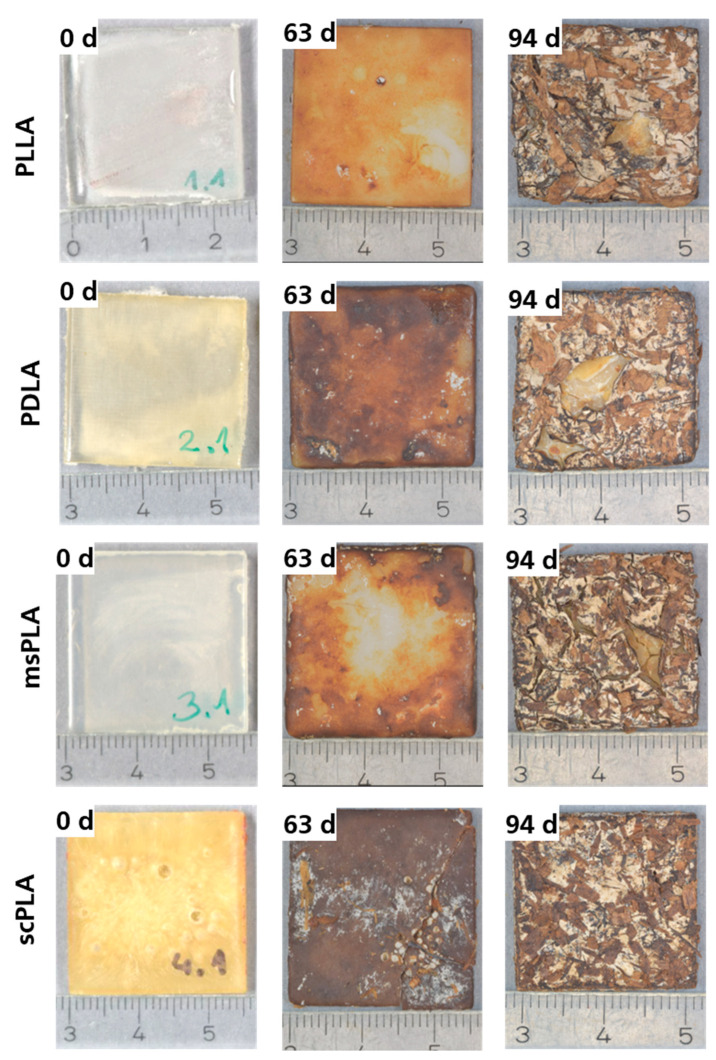
Photos of PLLA, PDLA, msPLA and scPLA samples stored in the soil after 0, 63 and 94 days. All samples were rinsed with distilled water.

After certain days, the samples were removed from the compost reactor and carefully washed with distilled water. Figure 5 shows the surface and dimension of PLLA, PDLA, msPLA and scPLA specimens at the starting point (0 days), after 63 days and after 94 days (end of composting). After 63 days, all samples have a rough surface, are cloudy and partially covered with a white film. The rough topography promotes the microorganism and water to penetrate the sample. For the sample msPLA, it can be clearly seen that the degradation process is starting from the outside and proceeds to the inside. After 94 days, the surface of all samples is strongly eroded, covered completely with a white film and merged with the soil. From a visual point of view, the sample scPLA shows the strongest surface erosion effect.

**Figure 6:**
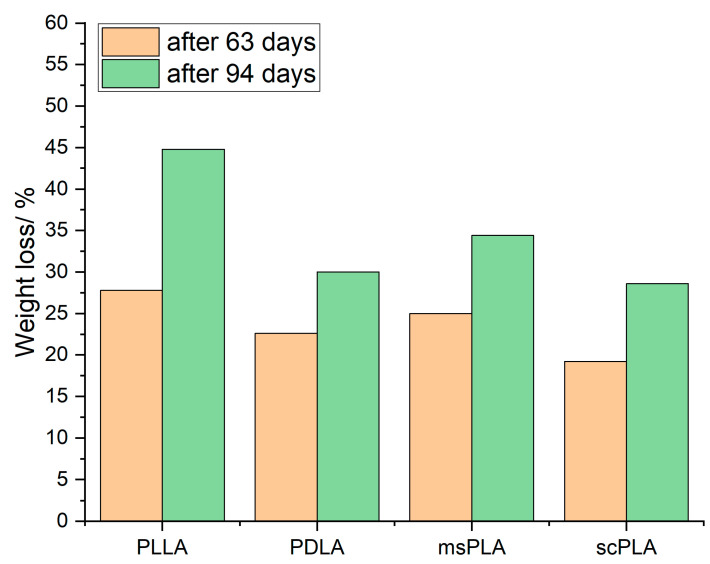
Weight loss after 63 days and 94 days for PLLA, PDLA, msPLA and scPLA.

Figure 6 shows the weight loss after 63 days and 94 days for PLLA, PDLA, msPLA and scPLA. For all samples, the greatest share in weight loss appears in the first half of the composting test. Whereas PLLA shows the highest weight loss with 27.8% after 63 days and 44.8% after 94 days, scPLA provides the lowest weight loss with 19.2% after 63 days and 28.6% after 94 days. These results are in good agreement with the crystallinity degree as PLLA hold the lowest crystallinity degree whereas scPLA has a high crystallinity. The amorphous domains tend to take up water at early decomposition times, initiating hydrolytic degradation before possible degradation begins in the crystalline regions.

The degree of disintegration (*D*) was calculated according to Equation (2) where *m*_0_ represents the original sample weight after drying and *m*_94_ the weight of the remaining sample after 94 days and drying:

(2) $$D=\frac{m_{0}-m_{94}}{m_{0}}\cdot 100\%$$

The values of *D* for the four tested materials can be seen in Table 3. With 44.8% for PLLA, 30.0% for PDLLA, 34.4% for msPLA and 28.6% for scPLA, PLLA holds the highest degree of disintegration.

**Figure 7:**
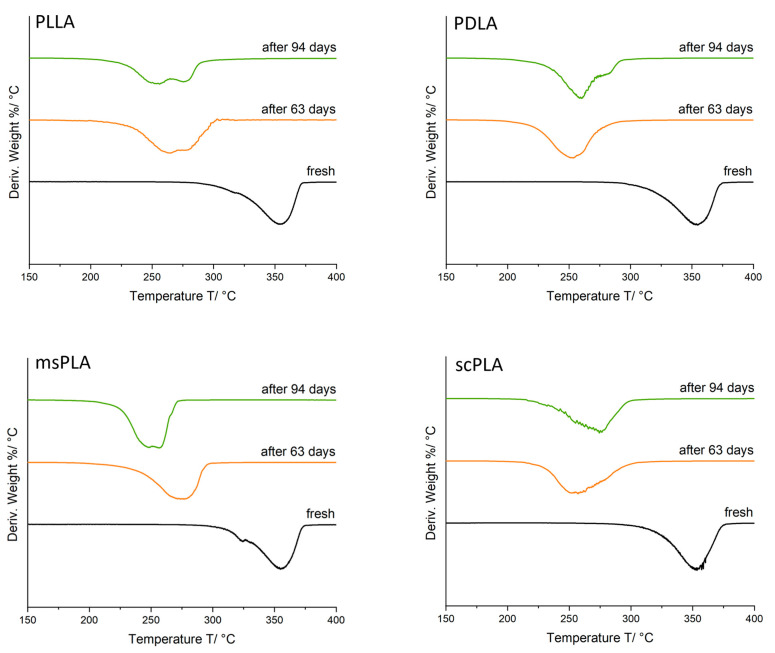
Deriv. weight curves of PLLA, PDLA, msPLA and scPLA after different decomposition times.

TGA was also performed for the samples after 63 days and 94 days under compost conditions. The derivative weight plots are shown in Figure 7. For all samples, the initial mass loss temperature and maximum loss rate temperature shifted to lower temperatures after 63 days of decomposition. The shift from 63 days to 94 days is not significant which is in accordance with the weight loss behavior (Figure 6a) and disintegration degree. It becomes visible with temperatures beside the main decomposition peak. This is a strong indication that upon composting, monomers and oligomers are formed that are less temperature stable. The strongest effect was obtained for PLLA and msPLA. Degradation processes are strongly dependent on the access of water and microorganisms into the sample [47]. Thus, the degradation rate decreases with an increase in crystallinity which is in accordance with the DSC results of PLLA, which show a low degree of crystallization whereas scPLA provides the highest crystallinity. The homocrystalline domains seem to be more accessible to hydrolysis than the stereocomplex regions [18]. scPLA shows the highest surface erosion but the lowest degree of disintegration; it seems that the stereocomplex content slows down the bulk erosion mechanism which is in line with the results presented by Kara et al. [18].

## 4. Conclusions

The present report addresses a material analysis of stereocomplex PLA (scPLA) for processing in melt spinning. scPLA is formed on a technical scale at a mass throughput of 2 kg/h based on PLLA and PDLA, both available on the market. The precipitated powder has a particle size of 493 µm. DSC and XRD measurements reveal the purity of the stereocomplex compared to semi-crystalline msPLA for fibers with textile strengths. The higher degree of crystallinity of 59.7% for scPLA compared to 50.5% for msPLA shows the potential for scPLA fibers with technical strengths. The rheology comparison of scPLA and msPLA shows that scPLA can be processed in melt spinning in principle. Nevertheless, the process must be adapted, e.g., during extrusion. Further research is needed here. The study of thermal stability shows that molecular weight is essential, whereas in the case of degradability, it can be seen that the degree of crystallinity and the crystalline structure are the essential factors.

Due to the now-sufficient availability, scPLA can be further processed in melt spinning on a technical scale in further research for developing scPLA fibers with technical strengths. This represents a major step towards transferring fibers made from biopolymers to the technical field. The expansion of the application area would have good consequences for both nature and the environment.

Possible applications in the technical field are, for example, ropes or composites: The scPLA fiber could replace the classic PP fiber used to make ropes [48]. Further properties of the scPLA fiber, such as the long-term behavior or UV stability, still need to be investigated. Self-reinforced composites could also be made from the scPLA material. For example, bicomponent fibers with semi-crystalline PLA in the sheath and scPLA in the core can be developed. After further processing into the composite, the semi-crystalline PLA forms the matrix and the scPLA the reinforcement. Improved mechanical properties are expected compared to the self-reinforced PLA made of amorphous PLA (matrix) and semi-crystalline PLA (reinforcement) [13].

## Figures and Tables

**Table 1:** Information of msPLA, PLLA and PLLA.

Name	Trade Name	D-Lactid Content	L-Lactid Content	*M_W_* (kg/mol)	*PDI* (-)
Starting materials for scPLA formation					
PDLA	Luminy D120	≥99	≤1%	150	1.6
PLLA	Luminy L130	≤1%	≥99%	170	2
Studied materials					
scPLA	scPLA formed of PDLA and PLLA				
msPLA	Ingeo 6100D	0.3%	99.7%	140	2

*M_W_* and *PDI* refer to molecular weight and polydispersity index, respectively. *M_W_* and *PDI* of PDLA [26] and PLLA [27] were taken from the literature, *M_W_* and *PDI* of msPLA were provided by NatureWorks.

**Table 2:** Properties (particle size, DSC, XRD and rheology) of msPLA and scPLA.

Measuring Method	Measured Variable	msPLA	scPLA
Particle size analysis	*S_M_* (µm)	Standard granules	493 ± 258
	*S_V_* (10%)		222
	*S_V_* (50%)		423
	*S_V_* (90%)		774
*S_M_* and *S_V_* refer to mean particle size and particle size at different percentile values, respectively.			
DSC	*T_C_* (°C)	116.1	-
	*ΔH_C_* (J/g)	41.4	-
	*T_M,HM_* (°C)	178.0	-
	*ΔH_M,HM_* (J/g)	47.0	-
	*X_C,HM_* (%)	50.5	-
	*T_M,SC_* (°C)	-	235
	*ΔH_M,SC_* (J/g)	-	87.2
	*X_C,SC_* (%)	-	59.7
*T_C_*, *∆H*, *T_M_* and *X_C_* refer to crystallization temperature, enthalpy, melting temperature and degree of crystallization, respectively.			
XRD	*2θ_HM_* (deg)	16.5; 18.5	-
	*2θ_SC_* (deg)	-	12.0; 20.5; 24.0
*2θ* refer to diffraction angle.			
Rheology	|η*| (Pa·s)	45–750	40–60
	*tan δ* (-)	4.5–45	9–26

|η*| and *tan δ* refer to complex viscosity and loss factor, respectively.

**Table 3:** Properties (TGA, degradation) of PLLA, PDLA, msPLA and scPLA.

Measuring Method	Measured Variable	PLLA	PDLA	msPLA	scPLA
TGA of the materials in granular/powder form	*T_5%_* (°C)	302.5	310	312.0	309.7
	*T_50%_* (°C)	347.1	348.0	348.9	348.2
	*T_95%_* (°C)	367.4	368.2	369.2	368.7
*T* refer to Temperature at different weight losses.					
Degradation	*W_0_* (g)	2.9	3.0	3.2	2.8
	*W_94_* (g)	1.6	2.1	2.1	2.0
	*D* (%)	44.8	30	34.4	28.6

*W* refers to weight loss at different days and D to degree of disintegration, respectively.

## Data Availability

The data presented in this study are available in this article “Properties of Stereocomplex PLA for Melt Spinning”.
